# Community-Led Data Collection: Enhancing Local-Level Scabies Surveillance in Remote Aboriginal Communities in Australia

**DOI:** 10.3390/tropicalmed8040200

**Published:** 2023-03-29

**Authors:** Miriam Glennie, Michelle Dowden, Meg Scolyer, Irene O’Meara, Geoffrey Angeles, Hannah Woerle, Patricia T. Campbell, Karen Gardner

**Affiliations:** 1 Public Sector Research Group, University of New South Wales, Canberra, ACT 2612, Australia; 2 One Disease, Darwin, NT 0811, Australia; 3 Peter Doherty Institute for Infection and Immunity, University of Melbourne, Melbourne, VIC 3010, Australia

**Keywords:** geohealth, active case detection, data ownership, neglected tropical diseases, scabies, community survey

## Abstract

Novel approaches to geohealth data analysis offer major benefits to neglected tropical disease control by identifying how social, economic and environmental elements of place interact to influence disease outcomes. However, a lack of timely and accurate geohealth data poses substantial risks to the accuracy of risk identification and challenges to the development of suitably targeted disease control programs. Scabies is one of many skin-related NTDs that is nominated as a priority for global disease control by the World Health Organization, but for which there remains a lack of baseline geospatial data on disease distribution. In this opinion paper, we consider lessons on impediments to geohealth data availability for other skin-related NTDs before outlining challenges specific to the collection of scabies-related geohealth data. We illustrate the importance of a community-centred approach in this context using a recent initiative to develop a community-led model of scabies surveillance in remote Aboriginal communities in Australia.

## 1. Introduction

Scabies is one of many skin-related NTDs that has been nominated as a priority for global disease control by the World Health Organization (WHO) [1], yet there remains a lack of baseline geospatial data on scabies distribution in many high-prevalence areas, including remote Australia. Caused by ectoparasite *Sarcoptes scabiei* var. *hominis*., scabies is primarily transmitted via skin-to-skin contact. Scabies causes skin lesions and itching, and is a risk factor for impetigo, acute rheumatic fever and chronic kidney disease [2].

Scabies is endemic in many remote Aboriginal communities in the Northern Territory (NT) of Australia. A severe and more infectious form of scabies, crusted scabies, is associated with high levels of morbidity [3]. Crusted scabies was made a notifiable disease by the NT government in 2016, and addressing the cycle of transmission between crusted and simple scabies is a significant component of prevention activity needed to reduce local-level prevalence [4]. However, neither crusted not simple scabies are the subject of regular, geospatial data capture to enable comprehensive surveillance.

A recent international framework for comprehensive scabies control proposed (1) mapping of disease burden; (2) delivery of interventions; and (3) establishing an appropriate monitoring and evaluation framework. Recommendations for standardised diagnostic and survey methods have been called for as a priority to identify target areas for community-level interventions via mass drug administration (MDA) [4]. However, there remains a lack of research or consensus on the design or implementation of appropriate surveys or in situ, community-level health data capture interventions [2]. Implementors of the standard models of community-based screen and treat campaigns have questioned the feasibility and sustainability of such strategies for scabies control at scale [5].

A geohealth approach would likely offer major benefits to scabies control by recognising the social, economic and environmental elements of place that interact to influence disease outcomes. However, careful consideration is necessary to inform the design of acceptable and effective data capture and management strategies for use in remote Aboriginal communities, and any approach must be community centred.

In looking at innovations in other skin-related NTDs, technological advances continue to improve the quality and quantity of remotely collected geospatial data, particularly climatic data important for spatial–epidemiological modelling of skin-related NTD distribution [6,7]. Yet, remotely designed data capture strategies can miss important local factors influencing scabies transmission. In NT communities, hygiene hardware such as washing machines, for example, or rates of household crowding, can be difficult to account for and may be overlooked. Stigma and lack of trust in external healthcare providers can influence participation in health screening. In addition to these challenges at the point of capture, inconsistency in national and/or centralized funding for scabies surveillance mean most communities remain passive recipients of sporadic interest in data collection and public health responses by central health authorities. These factors and others interact to perpetuate the lack of quality observational data for scabies surveillance, particularly health and social data collected in situ from under-served populations. These data limitations weaken the validity of geospatial modelling and impede accurate risk identification, making it difficult to develop suitably targeted interventions.

In this opinion paper, we draw on relevant literature and our experiences as providers and researchers in remote Aboriginal communities to consider implementation challenges in the collection of scabies-related geohealth data. We first look for lessons on impediments to geohealth data capture for other skin-related NTDs, before outlining the importance of a community-centred approach in this context. We illustrate—including how and why community health services should be at the centre of in situ data collection, with reflections on a recent initiative by One Disease—a not-for-profit organisation aiming to eliminate crusted scabies in the NT of Australia; this initiative sought to develop the community-level capability in geohealth data capture and scabies surveillance alongside community acceptability of data collection. The localized nature of skin-related NTDs and the social, economic and environmental factors contributing to transmission necessitate community-level data and responses [8]. We hope that our experiences and reflections contribute to knowledge and practice in community involvement in skin-related NTD surveillance.

## 2. Impediments to Skin-Related NTD Data Capture

NTDs are characterised by health system neglect [9] which is reflected in a lack of investment in capability and systems for detection. The communities in need of skin-related NTD control programs are under-resourced and under-served, which results in data gaps in addition to higher prevalence. This challenge is exacerbated by the stigma of skin-related NTDs, which limits healthcare seeking [10]. In these circumstances, active case detection involving community-based screening is often the only way to identify missed cases and to generate high-quality geospatial data capable of informing targeted interventions and control programs. However, active case detection (ACD) campaigns for skin-related NTDs often fail to develop local capability for data collection and fail to address stigma, limiting opportunities for more sustained data capture and surveillance.

### 2.1. Failure to Develop Local Capability for Data Collection

Most active case detection campaigns for skin-related NTDs involve prevalence surveys that are initiated and led by health authorities external to the community, e.g., [11,12,13]. Although often due to practicalities in resource-poor, high-population-density settings, standard ACD campaigns tend to be extractive—reflecting a ‘parachute science’ model of geohealth that is investigator led and involves limited community design input [14]. Local health workers are often employed to support implementation and community buy-in for screening, but community health service providers are rarely involved in campaign design or afforded data ownership.

This centralisation mirrors systems of data capture and ownership relating to passively (routinely) collected disease data. Problematically, the data collated via data sharing arrangements within and across health jurisdictions are often aggregated and retained by central health authorities without reporting back localised data to those involved in community health service delivery.

The exclusion of community health service providers from campaign design and data ownership reinforces local-level capability gaps in skin-related NTD surveillance and response. It can also result in immediate failure of ACD campaigns, as local providers may not be equipped to provide a public health response to an influx of newly detected cases. In prevalence surveys for leprosy, for example, there are examples of local health services not having the capacity to provide timely confirmatory diagnosis of suspected cases and not having sufficient supplies of the necessary multi-drug therapies [11,15]. In such circumstances, the failure to involve and resource community health service providers can not only limit the effectiveness of one-off detection campaigns, but can discourage future healthcare seeking and contribute to ongoing transmission and data gaps resulting from poor quality passive case detection data.

### 2.2. Failure to Address Stigma

Stigma is an important contributor to data gaps as well as the burden of skin-related NTDs. As skin-related NTDs mostly impact socially, culturally and economically marginalised communities [9], members of these communities experience multiple stigmas, as exclusion and discrimination create group stigma that compounds disease stigma [16]. Both individual and disease stigma have wide-ranging negative consequences for the social, economic and health outcomes of patients [17]. Stigma contributes to the risk of delayed detection in addition to data gaps; this exacerbates morbidity and mortality risks amongst infected individuals, as well as furthering disease transmission [13].

Research and data strategy on skin-related NTD detection frequently raises the need to mitigate stigma but seldom provides examples of how to do this, e.g., [10]. The absence of socially and culturally appropriate design in disease detection campaigns limits community trust and acceptability, which can manifest as refusal to accept screening or treatment [18,19]. Again, these failures can create negative repercussions beyond the immediate campaign by further reducing community trust in health services and reducing future health-seeking behaviour.

## 3. Overcoming Impediments to Data Collection for Scabies

Reflecting on the impediments identified in data capture strategies for other skin-related NTDs, the not-for-profit organisation One Disease sought to support the development of a community-led model in the NT. This approach enabled community health organisations to address their own data gaps on scabies and overcome impediments to data collection by developing internal capability via the design and implementation of a community-led local area scabies prevalence estimation survey. It was hoped that this would be the first in a model of regular active detection to enable ongoing community-level surveillance.

One Disease had been working in the NT since 2010, with the goal of eliminating crusted scabies. The organisation’s aims were to improve detection and treatment of crusted scabies, including scabies control to reduce risk of reinfection, and to reduce stigma to encourage individual health seeking. One Disease’s work reflected the importance of partnering with remote communities, state health and community health service providers to develop capabilities and address cross-boundary gaps in data and service provision.

In a recent initiative, One Disease sought to leverage its partnership approach to support a novel, community-led model that could serve as alternate to the parachute science approach to in situ data capture and an example of a community-led approach to scabies surveillance. In this initiative, One Disease commissioned epidemiological advice on prevalence estimation using community survey, treat and screen design. The organisation supported local community health services to translate methodological guidance into an actionable, culturally relevant and acceptable community survey.

Mitigating the risk of stigma was key to the translation of standard screening methodology to locally relevant and acceptable survey in this context. Australian Aboriginal and Torres Strait Islanders are serviced by a network of Aboriginal Community Controlled Health Organisations (ACCHOs). These community controlled and operated organisations are well equipped to understand the myriad sources of stigma relevant to scabies detection and to design campaigns that mitigate those risks. For example, the association between scabies and poverty-induced overcrowding has created wrongly held assumptions that scabies transmission results from poor personal hygiene. As the stigmatisation of scabies stems partly from this belief, sampling was identified as one of the most sensitive aspects of survey implementation. Selective sampling, including random sampling as was implemented in this survey, creates a risk of stigmatising visited households, as community members may assume those households are selected on the basis of poor personal hygiene or other associated markers of poverty.

To mitigate the risk of generating shame amongst visited households, it was essential to ensure that the random sampling technique was both well understood and widely understood. Methods that enable clear and public demonstration of a random sample, for example, publicly drawing household allotments or addresses from a hat, may help to validate claims that the selection was not based on personal characteristics or healthcare worker discretion, in addition to enabling the collection of geospatial markers for subsequent analysis.

A screen and treat approach, in which free pharmacological treatment (two doses of ivermectin 7 days apart) was provided immediately to all household members upon detection of scabies, was deemed appropriate. A community-level prevalence of over 10% could be used as an indicator to consider implementing an MDA [4].

In addition to supporting immediate geospatial data capture, local area scabies prevalence estimation and surveillance capability development, community-led surveillance models enable community ownership over localised geohealth scabies data. This may enable more timely public health responses by complementing and/or triggering centrally initiated disease control initiatives, while also enabling consent of data sharing to enable centralized geospatial epidemiological modelling. Such community-led models may be particularly well suited to settings with lower population density (making localised data capture more feasible), settings in which the local community is well represented in health service providers and in which health service providers are sufficiently resourced to have capacity for proactive health screening.

## 4. Discussion

In many contexts, establishing acceptable and sustainable NTD surveillance systems requires a shift away from parachute science that is extractive and investigator led to participatory action models that are co-produced with local communities. Enhancing local level surveillance to support comprehensive geohealth data capture and disease control, while enabling geospatial monitoring and analysis centrally, requires local-level capacity and ownership. Communities need to be at the centre of campaign design to mitigate risks of stigma-generating actions and to contribute community knowledge related to person-to-person transmission and other factors affecting data collection, feedback and access to treatment.

Information feedback loops to support quality improvement at the individual health service level are also needed to ensure geographically localized data are used in ongoing monitoring activities and linked to treatment provision. In Australia, the Aboriginal Community Controlled sector has well-developed quality improvement processes in place that are supported by the state-based peak body. The knowledge and expertise of this sector is essential to effective skin-related NTD control in Australia.

Failing to report back results from prevalence surveys or other in situ data capture methods is a risk of parachute science approaches that must be avoided. Many high-risk and under-served communities remain captive to sporadic interest from central health authorities’ interest in NTD mapping and do not ‘own’ the data relevant to their own community.

Despite the value of enhancing local-level surveillance capability, it is important to acknowledge that data alone are insufficient to substantially reduce the burden of skin-related NTDs. Disease control efforts cannot be separated from the social determinants of health which produce them. Worldwide, transmission of scabies is underpinned by overcrowding and poor living conditions [20,21]. In the NT, studies have shown that mobility between communities, limited opportunities for privacy in overcrowded households [22,23] and poor housing hardware [24] are major barriers to ongoing prevention and treatment of skin diseases. Continued innovations in geohealth data collection and monitoring are essential to advance multifaceted, socially embedded and culturally informed disease control programs.

## Data Availability

No new data were created or analyzed in this study. Data sharing is not applicable to this article.
